# Patient delay is the main cause of treatment delay in acute limb ischemia: an investigation of pre- and in-hospital time delay

**DOI:** 10.1186/1749-7922-9-56

**Published:** 2014-11-05

**Authors:** Louise S Londero, Birgitte Nørgaard, Kim Houlind

**Affiliations:** Department of Cardiothoracic- and Vascular Surgery, University Hospital of Odense, Sdr. Boulevard 29, 5000 Odense C, Denmark; Institute of Public Health, University of Southern Denmark, Sdr. Boulevard 29, 5000 Odense C, Denmark; Department of Vascular Surgery, Kolding Hospital, Skovvangen 2, 6000 Kolding, Denmark

**Keywords:** Acute limb ischemia, ALI, Treatment delay, Fast track department, Diagnostic packages, Patient delay

## Abstract

**Background:**

The prognosis of acute limb ischemia is severe, with amputation rates of up to 25% and in-hospital mortality of 9-15%. Delay in treatment increases the risk of major amputation and may be present at different stages, including patient delay, doctors´ delay and waiting time in the emergency department. It is important to identify existing problems in order to reduce time delay.

The aim of this study was to collect data for patients with acute limb ischemia and to evaluate the time delay between the different events from onset of symptoms to specialist evaluation and further treatment with focus on pre-hospital and in-hospital time delays.

**Methods:**

We conducted a prospective cross-sectional cohort study including all patients suspected with acute limb ischemia who were admitted to the emergency department of a community hospital in a six months period. Temporal delay in the different phases between the time of occurrence of symptoms and completion of treatment was recorded prospectively. All patients who underwent intervention had a 30 days follow-up with regard to major amputation of the leg and survival.

**Results:**

A total of 42 patients (21 men and 21 women) age 73 (20–95) years (median (range)) was identified.

From onset of symptoms to first contact with a doctor the time for all patients were 24 (0–1200) hours. Thirty patients needed immediate intervention. In the group of fourteen patients who had immediate operation, the median time from vascular evaluation to revascularization was 324.5 (122–873) minutes and in the group of eight patients that went through an imaging procedure before an operation the median delay was 822 (494–1185) minutes from specialist assessment to revascularization. The median time for revascularization among four patients, who were treated with arterial thrombolysis was 5621 (1686–8376) minutes.

At 30 days follow up, six patients had had the ischemic limb amputated above the ankle and four patients had died.

**Conclusions:**

We found that the largest time delay was between onset of symptoms and first contact to a medical doctor. A greater public awareness is needed, so as to facilitate urgent revascularisation and improve outcomes.

## Background

Acute limb ischemia (ALI) is a sudden decrease in limb perfusion causing a potential threat to the limb viability [1]. The event may be caused by thrombosis, embolism, peripheral aneurysm with embolus/thrombosis, acute graft occlusion, iatrogenic intervention or by trauma. The prognosis is severe, with reported amputation rates of up to 25% and in-hospital mortality of 9-15% [1–3]. Delay in treatment of acute lower limb ischemia increases the risk of amputation [4–7].

The delay may be present at different stages, including patient delay, delay caused by the referring medical doctor, waiting time in the emergency department, waiting time for diagnostic imaging, or time for preparation at the operating theatre, including anaesthesia. Before creating strategies for reducing treatment delay, existing problems need to be identified.

It has been shown that implementation of ED fast track systems reduces ED waiting time and overcrowding [8–10]. In Denmark, the fast track organisation of Emergency Departments (ED’s) has established length of stay time targets [11, 12] similar to those known in the UK and Australia [13, 14]. This has been done to ensure a punctual evaluation, examination and initiation of treatment of the patient including specialist examination within 30 minutes from arrival and a diagnosis within four hours. To optimize the patient flow 36 diagnostic packages have been prepared. These are based on main symptom with respect for triaging and time sensitive diagnosis. One of the packages is “Pain in extremity” and includes ALI [11]. The implementation in our hospital was done in September 2012. It is, however, unknown whether other causes for time delay are equally or more important for patients suspected with ALI.

The aim of this study was to collect data for patients with ALI and evaluate the time delay between the different events from onset of symptoms to specialist evaluation and further treatment with focus on pre-hospital and in-hospital time delays.

## Methods

### Design

We conducted a prospective cross-sectional cohort study. The study was approved by the Danish Data Protection Agency. Data collection took place from 01.10.12 to 31.03.13, at Kolding Hospital, a community hospital with a department for vascular surgery with a catchments area covering both suburban and rural districts.

### Settings

The patients´ first contact with the medical care system is with a non-vascular specialist outside the hospital. When ALI is suspected, the non-vascular specialist consults a specialist in vascular surgery and the patients are admitted to the hospital. The ED fast track unit is notified by the vascular surgeon. All patients suspected with ALI are admitted to the fast track area in the ED.

The fast track unit is separated from the rest of the ED. It is open 24 hours a day and has its own nurses and staff station. There are two senior doctors, one specialist in internal medicine and one specialist in surgery, in the department from 08.00 am to 12.00 pm and at night they are on call. Also there are a number of junior doctors associated with unit.

The vascular specialist is summoned to the ED when the patient suspected with ALI arrives and will be the first doctor to see the patient within the ED fast track area.

### Data collection

For each patient admitted, the specialist vascular surgeon on call started the data collection of the particular patient. A registration form was filled in with information about gender, age and earlier vascular procedures. Furthermore, onset of symptoms, first contact with a medical doctor (a non-vascular specialist), time for referral to the vascular department, time for arrival and time for vascular evaluation were registered. All patients were scored according to the classification suggested by Rutherford, where the severity of ALI is divided into four categories [6]. In class 1 the limb is not immediately threatened and is considered as subacute ischemia, while in class 2A and 2B the limb is threatened and needs immediate revascularization. Patients with class 3 ischemia have irreversible damage and there is no indication to improve the blood supply.

If the patient was not judged to have ALI the registration was stopped after the vascular examination. If they had ALI and needed further intervention, time was registered for the beginning and end of the procedure. Further interventions were computed tomography (CT) or magnetic resonance (MR)-imaging, operation, endovascular procedure or amputation.

When the patient arrived to the ED, examination of the leg was used to define the severity of the ischemia. ALI was classified into one of four categories based on clinical findings and Doppler measurements.

All patients who underwent intervention had a 30 days follow-up with regard to major amputation of the limb and death.

### Analyses

For each patient the time between the registrations, in a chronological order, was measured. Descriptive statistics were performed using Microsoft Excel Version 7.0. To compare patient related time delay in patient with and without prior vascular surgery we performed Wilcoxon rank sum test using Stata, (Version 13.1, StataCorp LP, College Station, Texas).

## Results

**Figure 1:**
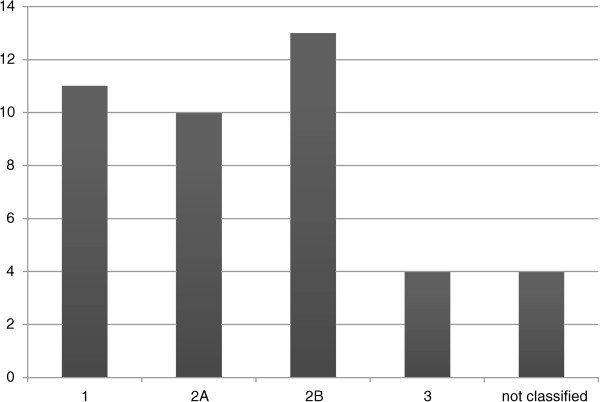
**Distribution of patients.** The distribution of patients (n = 42) according to levels of severity of acute limb ischemia.

A total of 42 patients (21 men and 21 women) ages 73 (20–95) years (median (range) were admitted on suspicion of ALI. Twenty-one patients (50%) had a prior history of vascular surgery for periphery arterial disease (PAD) in one or both legs. Twelve patients did either not have ALI or had ALI class 1, which did not need immediate intervention, and registration stopped after vascular examination. Of these patients three had deep venous thrombosis (DVT), one had neuropathic pain, seven had ALI class 1 and one had class 2A ischemia, but it was found that no immediate treatment was required. The distribution of patients according to levels of severity of acute limb ischemia is shown in Figure 1.

### Time from onset to specialist assessment (n = 42)

**Figure 2:**
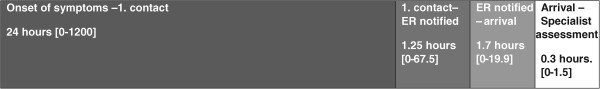
**Pre-hospital time delay.** The time delay from onset of symptoms to specialist assessment (median time [range]) for all 42 patients suspected with ALI.

For all patients the time from onset of symptoms to specialist assessment the median time was 27.25 (1.65 – 1202.5) hours. This time period was divided into smaller time periods as showed in Figure 2.

Median time from onset of symptoms to first contact with a medical doctor was 24 (0–1200) hours. For patients with a prior history of vascular surgery the median time was 22 (0–240) hours and for patients with no earlier history of vascular surgery the time was 26.35 (0.17- 1200) hours. The time difference between the two groups was not significant (p = 0.4).

The time from first contact to a doctor until the ED was notified was 1.25 (0–67.5) hours and transportation time to the ED was 1.7 (0–19.9) hours. Median time from arrival to the ED to evaluation by a vascular specialist was 0.33 (0–1.5) hours. A total of 26 patients (62%) were assessed by the vascular surgeon on call within 30 minutes after arrival, 9 (21%) within an hour, and 7 (17%) waited for more than one hour.

### ALI classified patients (n = 30)

**Figure 3:**
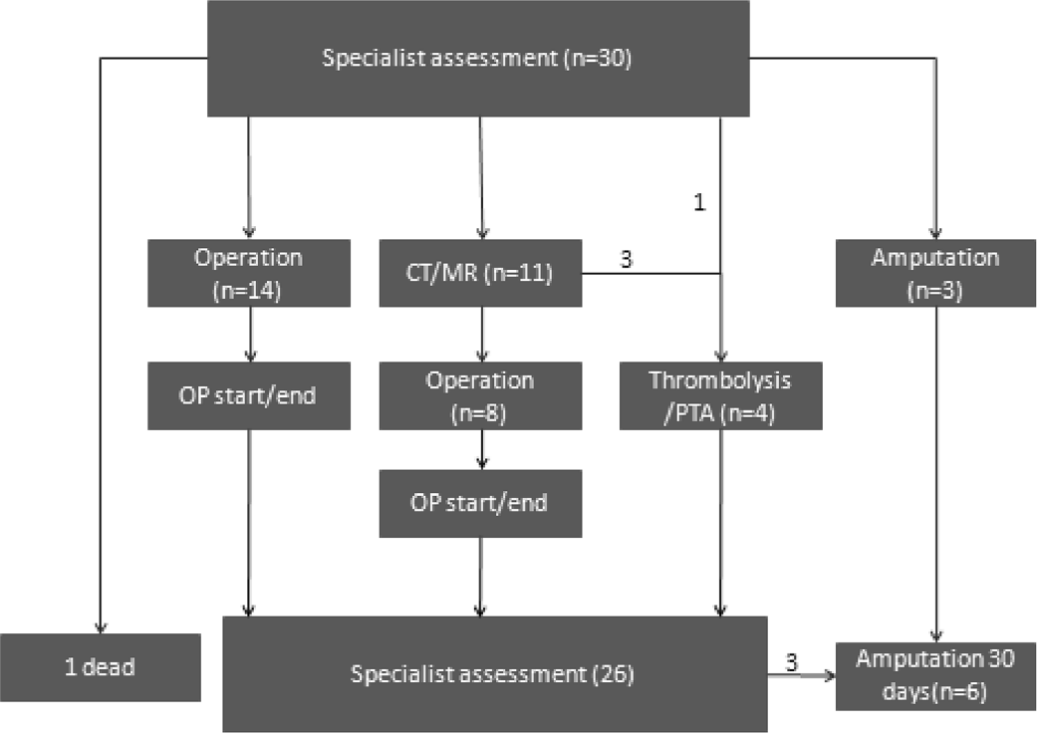
**From specialist assessment to revascularization.** The flowchart shows the distribution of the 30 patients, in need of acute intervention after specialist assessment.

Thirty patients were classified with ALI and needed immediate intervention as seen in Figure 3. Fourteen patients needed operation immediately, 11 patients went through diagnostic imaging before further intervention, one patient went directly to diagnostic angiography and thrombolysis and for 3 patients the ischemia was so severe, that revascularization was not indicated; they went directly for an amputation of the limb. One patient was classified with severe ALI in both legs caused by an occlusion of the aorta in the setting of a major myocardial infarct and died before any action could be taken.

Among the 14 patients operated immediately, it was possible to restore blood flow to the limb in 12. Eight of the 11 patients who underwent imaging had an operation afterwards; each having successful revascularization, yet two patients had re-operation due to new occlusions. Three patients underwent thrombolysis and one had an endovascular procedure, all of them resulting in restored blood flow.

**Figure 4:**
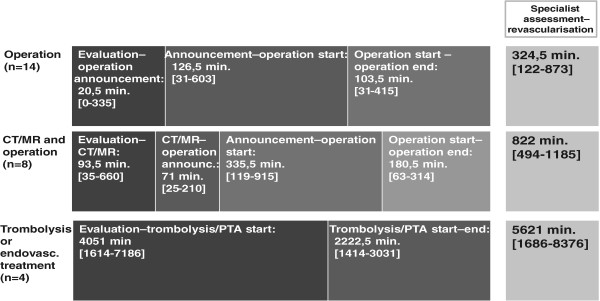
**In-hospital time delay.** The in-hospital time delay for all three groups with vascular intervention.

In the group of patients who had immediate operation, 10 had an embolectomy, two had a thrombendarterectomy and two had peripheral bypass operation. The median time from vascular evaluation to revascularization (defined as time to end of successful surgery) was 324.5 (122–873) minutes, whereas in the group of patients that went through an imaging procedure before operation the median delay was 822 (494–1185) minutes from vascular evaluation to revascularization. The median time for revascularization in the thrombolytic group was 5621 (1686–8376) minutes Figure 4.

### 30-days follow-up on amputation and death

At 30 days follow up, six patients had had the ischemic limb amputated above the ankle; four of these amputations were above the knee. Three of the patients were amputated without prior attempt of revascularization. In the amputation group (n = 6) the median time from onset of symptoms to arrival at the ER was 68.8 (24–1202) hours. The patient who did not arrive until 1202 hours (50 days) after onset of symptoms was an 83 years old man suffering from a neurodegenerative disease. He was referred acutely, without being able to tell for how long he had had symptoms. The hospital files showed that he had been seen in the emergency department 50 days earlier with complaints about pain in the leg. At that time, it was suspected to be muscle or join discomfort and he was released without seeing a vascular specialist. Fifty days later his family doctor was called to see him and he referred the patient to the hospital suspecting ALI.

Five out of the six patients who were amputated were assessed by a vascular specialist within 30 minutes after arrival; one waited 90 minutes. Three of these patients had an attempt of surgical revascularization. From vascular specialist assessment to revascularization it took 122, 365 and 620 minutes respectively. Two of the patients had an amputation above knee within 24 hours after attempt of revascularization and the last one had an amputation below knee after 9 days.

Four patients died within 30 days after referral to the vascular department, two had had a revascularization procedure that failed to restore blood flow, one had an amputation below the knee and the last one died of myocardial infarct shortly after arrival to the emergency department.

## Discussion

Patients with acute limb ischemia have a poor prognosis with regard to major limb amputations and death. The rate of major amputations is reported to be up to 25%, including both non-salvageable limbs and limbs thought to be salvageable [1]. For patients with attempt of revascularization the amputation rate is 9-15% [1–3]. In our study six out of 30 (20%) patients found to have ALI underwent major amputation within the first 30 days, three of them had non-salvageable limbs at the time of vascular specialist assessment and no revascularization procedure was attempted.

Eliason and colleagues reported 9.3% in-hospital mortality for patients admitted with ALI [2], while Kuukasjärvi reported a 30 days mortality of 13% [15]. This is comparable to our findings where the 30 day’s mortality rate was 13.3% (four out of 30).

There is a relationship between the delay from onset of symptoms to revascularisation and subsequent mortality or limb loss. As an example, Morris-Stiff and colleagues recently compared the results after peripheral arterial embolectomy from two different historical time periods in the same community hospital [16]. In both time periods, patients with an interval between symptoms and treatment of less than five hours had a more favourable outcome than those who suffered a longer delay. Therefore, it is of great importance that patients with suspected ALI are referred to a hospital with vascular specialists immediately.

In our study the largest pre-hospital time delay occurred between the onset of symptoms and the first contact to a doctor, this was the case for all patients, including the ones with irreversible ischemia. The median time was 24 hours, which is similar to findings in earlier studies [7, 16]. Burgess and colleagues found that mean delay from onset of symptoms to first contact to a doctor was 29 hours and the main reason for delay was the patient. Also, Morris-Stiff and colleagues found that despite of advances in improvements in pre- and peri-operative management of ALI, the amputation rate was not significantly reduced and, therefore, concluded that the main reason was the fact that the patients still had a mean time from symptoms to revascularisation of 24 hours [16].

Patients with a prior history of vascular surgery for PAD tended to respond faster to symptoms than patients without prior history, suggesting that the first group is more aware of the importance of symptoms. This difference was, however, not statistically significant. Time delay between the first contact to a doctor and the arrival at the emergency department was reasonable taking into consideration that most patients were seen and examined by the non-vascular specialist outside the hospital before referral. To our knowledge, this is the first study evaluating the different time steps from onset of symptoms to revascularisation.

When looking at the in-hospital delay one of the main goals in the fast track program is to have the patients evaluated by a specialist within 30 minutes after arrival. In our study 62% of the patients were evaluated within 30 minutes, which leaves place for improvement. On the other hand, only 17% waited for more than one hour due to the fact that the surgeon was occupied with other acute tasks. Whether the fast track programme has shortened the in-hospital time delay is not certain, because our data collection started after the implementation.

A bit surprisingly we found that patients who went for imaging before further intervention had an almost 8.5 hours longer in-hospital delay than patients who needed immediately operation. This was mainly due to waiting time for the CT- or MR-imaging. Though imaging is considered a part of the fast track programme and should be done within the first four hours after arrival. Local changes have now been done in order to meet this goal of the fast track programme. We recognize limitations of the study, including especially a small sample size and also the fact that we only studied practice in one hospital. The relative importance of each of the different phases of in hospital delay cannot be expected to be the same in other locations, although the method used may be thought of as an inspiration for local analyses in other hospitals. However, the primary finding; that patient delay is the main reason for treatment delay in ALI, seems to be consistent with findings from other geographical areas and other time periods [7, 16] and may be a finding of general interest.

## Conclusions

In summary, we found that the main component of treatment delay is the time between onset of symptoms and the first contact to a doctor. Although we have not specifically asked the patients about the reasons for this delay, it seems safe to suggest that greater public awareness is needed, so as to facilitate urgent revascularisation and improve outcomes. Public campaigns, stressing the need for urgent treatment of ALI, might be a tool to improve results. In order to avoid misdiagnosing patients with ALI it may be helpful if all patients with acute pain in a leg, not caused by fractures or other obvious causes, are seen by a vascular specialist when referred to an emergency department according to the fast track organisation [11]. We recommend further studies including more centres and a larger number of patients in order to produce valid and generalizable results. We also recommend studies investigating the specific reasons for delay from onset of symptoms and the first contact to a doctor in order to minimize this delay and thereby improve outcomes for ALI patients.

## Consent

According to Danish legislation and The National Committee on Health Research Ethics written consent from the patients is not required.
